# Phase 1 study of pembrolizumab plus chemotherapy in Japanese patients with extensive-stage small-cell lung cancer

**DOI:** 10.1007/s10637-023-01411-1

**Published:** 2024-02-01

**Authors:** Naoyuki Nogami, Takaaki Tokito, Yoshitaka Zenke, Miyako Satouchi, Takashi Seto, Hideo Saka, Junko Ohtani, Shirong Han, Kazuo Noguchi, Makoto Nishio

**Affiliations:** 1 Department of Community Medicine, Pulmonology and Cardiology, Ehime University Graduate School of Medicine, 454 Shitsukawa, Toon, Ehime 791-0295 Japan; 2 Division of Respirology, Neurology, and Rheumatology, Department of Internal Medicine, Kurume University School of Medicine, Kurume, Japan; 3 Department of Thoracic Oncology, National Cancer Center Hospital East, Kashiwa, Japan; 4 Department of Thoracic Oncology, Hyogo Cancer Center, Akashi, Japan; 5 Department of Thoracic Oncology, National Hospital Organization Kyushu Cancer Center, Fukuoka, Japan; 6 Department of Respiratory Medicine, National Hospital Organization Nagoya Medical Center, Nagoya, Japan; 7 MSD K.K., Tokyo, Japan; 8 Department of Thoracic Medical Oncology, The Cancer Institute Hospital of Japanese Foundation for Cancer Research, Tokyo, Japan

**Keywords:** Pembrolizumab, Immunotherapy, Small-cell lung cancer, Platinum

## Abstract

**Background:**

Part E of the KEYNOTE-011 (NCT01840579) study assessed the safety and antitumor activity of pembrolizumab plus platinum-etoposide chemotherapy in Japanese patients with previously untreated extensive-stage small-cell lung cancer (ES-SCLC).

**Methods:**

Patients received 4 cycles of pembrolizumab (200 mg) every 3 weeks in combination with cisplatin (75 mg/m^2^) and etoposide (100 mg/m^2^; days 1, 2, 3) in cohort 1; with carboplatin (AUC 5 mg/mL/min) and etoposide (100 mg/m^2^; days 1, 2, 3) in cohort 2; or with cisplatin/etoposide and pegfilgrastim (3.6 mg; cycle 1, day 4) in cohort 3. Combination therapy was followed by pembrolizumab monotherapy (31 cycles). The primary endpoint was safety and tolerability (including dose-limiting toxicities; DLTs).

**Results:**

Fifteen patients were included in the study (cohort 1, n = 6; cohort 2, n = 6; cohort 3, n = 3). Median time from treatment allocation to data cutoff was 22.1 months (range, 4.1‒32.4 months). DLTs occurred in 3 patients in cohort 1 (one patient with grade 4 laryngeal stenosis and grade 3 febrile neutropenia; two patients with grade 3 febrile neutropenia); no patients in cohorts 2 or 3 experienced DLTs. Grade ≥ 3 treatment-related adverse events included leukopenia (67%) and neutropenia (87%). Among all patients, ORR was 67% (95% CI, 38%‒88%) and median DOR was 4.5 months (range, 2.8‒28.8 months). Median PFS was 4.2 months (95% CI, 3.0‒7.8 months) and median OS was 22.1 months (95% CI, 7.4‒25.9 months).

**Conclusion:**

Pembrolizumab in combination with platinum-etoposide therapy had manageable toxicity with no new safety signals and was associated with antitumor activity in Japanese patients with ES-SCLC.

**Trial Registration:**

ClinicalTrials.gov, NCT01840579.

**Supplementary Information:**

The online version contains supplementary material available at 10.1007/s10637-023-01411-1.

## Introduction

Lung cancer is the leading cause of cancer death in Japan; in 2020, it was estimated to account for 13% of new cancer diagnoses and 20% of cancer deaths [1]. Based on a registry study of patients with lung cancer receiving medical treatment, small-cell lung cancer (SCLC) accounts for 19% of all lung cancers in Japan [2]. Until recently, first-line treatment for patients with extensive-stage (ES) SCLC was typically platinum-based chemotherapy with etoposide [3]. Despite such treatments, outcomes for patients with SCLC have been very poor: median overall survival (OS) is approximately 7 months, and the 5-year survival rate is approximately 6% [4].

The programmed cell death protein 1 (PD-1) pathway is frequently altered in cancer, leading to inhibition of active T-cell‒mediated immune surveillance of tumors. Consequently, PD-1 and its ligands (programmed death ligand 1 and 2 [PD-L1 and PD-L2]) have been the targets of anticancer immunotherapies [5–7]. SCLC is associated with heavy smoking and a high mutation burden [8], suggesting that such tumors may be immunogenic and that immunotherapies may be effective in the treatment of SCLC. Immunotherapies that block PD-1 pathway signaling have been shown to improve outcomes in patients with ES-SCLC. In the IMpower133 trial, adding the anti‒PD-L1 monoclonal antibody atezolizumab to platinum-etoposide chemotherapy resulted in improved OS (hazard ratio [HR], 0.70; 95% CI, 0.54‒0.91; *P* = 0.007) and progression-free survival (PFS; HR, 0.77; 95% CI, 0.62‒0.96; *P* = 0.02) compared with placebo plus platinum-etoposide [9]. In the CASPIAN trial, hazard ratios for OS (0.73; 95% CI, 0.59–0.91; *P* = 0.0047) and PFS (0.78; 95% CI, 0.65–0.94; statistical significance not evaluated) both favored treatment with the anti‒PD-L1 monoclonal antibody durvalumab plus platinum-etoposide compared with placebo plus platinum-etoposide [10].

Pembrolizumab, a humanized anti‒PD-1 monoclonal antibody, blocks the interaction between PD-1 and its ligands PD-L1 and PD-L2. Pembrolizumab monotherapy showed antitumor activity in patients with previously treated ES-SCLC in the KEYNOTE-028 and KEYNOTE-158 studies [11–13]. In a pooled analysis of patients from KEYNOTE-028 and KEYNOTE-158, the objective response rate (ORR) was 19% [13]. Addition of pembrolizumab to platinum-etoposide chemotherapy in the phase 3 KEYNOTE-604 study significantly prolonged PFS compared with placebo plus chemotherapy (HR, 0.75; 95% CI, 0.61‒0.91; *P* = 0.0023); however, OS was not significantly improved (HR, 0.80; 95% CI, 0.64‒0.98; *P* = 0.0164) [14].

KEYNOTE-011 (NCT01840579) is an open-label, multipart, phase 1 study of pembrolizumab in a Japanese population of patients with advanced solid tumors. We report results from part E, which assessed the safety and antitumor activity of pembrolizumab in combination with standard platinum-etoposide chemotherapy in Japanese patients with ES-SCLC.

## Methods

### Patients

Patients were eligible for inclusion in part E of the KEYNOTE-011 study if they were ≥ 20 years of age with previously untreated, histologically or cytologically confirmed ES-SCLC (stage IV or T3‒4 due to presence of multiple lung nodules per the American Joint Committee of Cancer, 7th edition), an Eastern Cooperative Oncology Group (ECOG) performance status of 0 or 1, and ≥ 1 radiographically measurable lesion per Response Evaluation Criteria in Solid Tumors (RECIST) version 1.1 (defined as a lesion ≥ 10 mm in the longest diameter or lymph node ≥ 15 mm in the short axis). Patients were required to have adequate organ function, including hematologic, renal, hepatic, endocrine, and coagulation laboratory values. Patients were excluded from the study if they had radiation therapy, chronic systemic steroid therapy, or any other immunosuppressive therapy ≤ 2 weeks before the first dose of study treatment. Also excluded were patients who had not recovered from adverse events (AEs) due to previous treatment > 4 weeks prior to the first dose of study treatment; had a history of acute diverticulitis, intra-abdominal abscess, gastrointestinal obstruction, or abdominal carcinomatosis; had a history of hematologic malignancy, primary brain tumor or sarcoma, or another primary solid tumor unless they had received potentially curative therapy with no evidence of disease for ≥ 5 years; had known active central nervous system metastases and/or carcinomatous meningitis (patients could participate in the study if they were clinically stable for at least 2 weeks with no evidence of new or enlarging brain metastases and were off steroids 3 days before dosing with study medication); had prior therapy with an anti‒PD-1, anti‒PD-L1, or anti‒PD-L2 agent or an antibody targeting other immune-regulatory receptors or mechanisms; or had participated in a study of an investigational agent or device within 30 days of study treatment.

### Study design

Patients received up to 4 cycles of intravenous pembrolizumab (200 mg on day 1 of each cycle) every 3 weeks (Q3W) in combination with intravenous cisplatin (75 mg/m^2^ on day 1) and etoposide (100 mg/m^2^ on days 1, 2, and 3) in cohort 1; with intravenous carboplatin (area under the curve 5 mg/mL/min on day 1) and etoposide (100 mg/m^2^ on days 1, 2, and 3) in cohort 2; or with intravenous cisplatin (75 mg/m^2^ on day 1) and etoposide (100 mg/m^2^ on days 1, 2, and 3) with prophylactic subcutaneous pegylated granulocyte colony-stimulating factor (G-CSF; pegfilgrastim; 3.6 mg on day 4 of cycle 1) in cohort 3. Combination therapy was followed by pembrolizumab monotherapy (200 mg Q3W for up to 31 cycles). Treatment was discontinued upon completion of 2 years of study treatment, documented disease progression, or unacceptable AEs.

### Endpoints

The primary endpoint was safety and tolerability of pembrolizumab, including dose-limiting toxicities (DLTs), AEs, and laboratory tests. DLTs were evaluated at completion of the DLT evaluation period in each patient (first 3 weeks after initiation of study treatments). The following events were considered DLTs if assessed as related to treatment by the investigator: grade 4 neutropenia lasting > 7 days, grade 3 or 4 febrile neutropenia (absolute neutrophil count < 1000/mm^3^ with single temperature > 38.3 °C or a sustained temperature ≥ 38 °C for > 1 h; for grade 4, life-threatening consequences and urgent intervention indicated), grade 4 thrombocytopenia (< 25,000/mm^3^), grade 4 anemia, grade 4 non-hematologic toxicity, grade 3 non-hematologic toxicity lasting > 3 days despite optimal supportive care, and any grade 3 non-hematologic laboratory values if medical intervention was required to treat the patient or the abnormality persisted for > 7 days. The target DLT rate was 30% using a toxicity profile interval design [15]. Exploratory endpoints included ORR, defined as the proportion of patients with a complete response (CR) or partial response (PR); duration of response (DOR), defined as the time from first evidence of CR or PR to disease progression or death; PFS, defined as the time from treatment allocation to the first of disease progression or death; and OS, defined as the time from treatment allocation to death.

### Assessments

All toxicities were graded using National Cancer Institute Common Terminology Criteria for Adverse Events, version 4.0. AEs were reported from the time of treatment allocation through 30 days following cessation of treatment; serious AEs were collected for up to 90 days after cessation of treatment or 30 days after cessation of treatment if the patient initiated new anticancer therapy, whichever was earlier.

Tumor imaging by computed tomography (CT) or magnetic resonance imaging (MRI) was done at baseline, every 6 weeks for the first 24 weeks, then every 9 weeks thereafter. Antitumor activity was evaluated based on CT or MRI per RECIST version 1.1 by investigator assessment. Patients who experienced progressive disease (PD) or started another treatment were followed for survival every 2 months from the last contact in the study.

### Statistics

Between 3 and 9 patients were planned to be enrolled in each cohort. Patients who received ≥ 1 dose of study treatment were included in the analysis. Data were summarized using descriptive statistics. DLT-evaluable patients had a DLT in the DLT evaluation period or had received ≥ 90% of the prescribed dose of pembrolizumab and the combination regimen and completed all safety evaluations in the DLT evaluation period without experiencing a DLT. DOR, PFS, and OS were summarized using the Kaplan-Meier method.

## Results

A total of 15 patients were enrolled in part E of the KEYNOTE-011 study between March 10, 2017, and February 28, 2020 (data cutoff): 6 patients in cohort 1 (pembrolizumab plus cisplatin and etoposide), 6 patients in cohort 2 (pembrolizumab plus carboplatin and etoposide), and 3 patients in cohort 3 (pembrolizumab plus cisplatin, etoposide, and prophylactic pegfilgrastim). Database lock was June 11, 2020.

Patient characteristics are summarized in Table 1. Median age was 63.5 years in cohort 1, 65.0 years in cohort 2, and 57.0 years in cohort 3. Overall, median age was 64 years, and approximately half of patients included in the study were men. Three patients in cohort 1, 1 patient in cohort 2, and 1 patient in cohort 3 had brain metastases at baseline.

**Table 1:** Patient demographics and baseline clinical characteristics

**Characteristic**	**Cohort 1** **Pembrolizumab Plus Cisplatin/Etoposide** **(n = 6)**	**Cohort 2** **Pembrolizumab Plus Carboplatin/Etoposide** **(n = 6)**	**Cohort 3** **Pembrolizumab Plus Cisplatin/Etoposide Plus Pegfilgrastim** **(n = 3)**	**Total** **(N = 15)**
Age, y				
Median	63.5	65.0	57.0	64.0
Range	60‒70	51‒80	55‒70	51‒80
Male	3 (50)	4 (67)	1 (33)	8 (53)
ECOG PS				
0	2 (33)	2 (33)	3 (100)	7 (47)
1	4 (67)	4 (67)	0	8 (53)
Former/current smoker	5 (83)	5 (83)	2 (67)	12 (80)
Metastatic stage				
M1	0	1 (17)	0	1 (7)
M1A	0	1 (17)	1 (33)	2 (13)
M1B	6 (100)	4 (67)	2 (67)	12 (80)
Brain metastases	3 (50)	1 (17)	1 (33)	5 (33)
Mean ± SD baseline tumor size, mm	149.6 ± 70.5	143.6 ± 77.7	64.4 ± 17.6	130.2 ± 71.7

All data are n (%) unless otherwise noted

*ECOG PS* Eastern Cooperative Oncology Group performance status

**Fig. 1:**
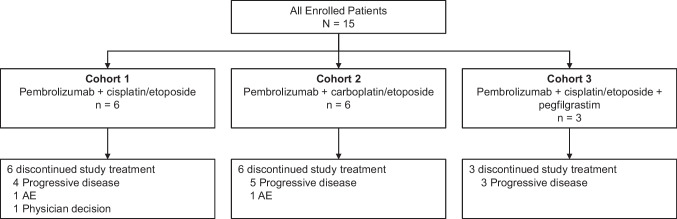
Disposition of patients in the study. AE, adverse event

Median time from treatment allocation to data cutoff (February 28, 2020) was 16.1 months (range, 4.1‒31.4 months) for cohort 1, 17.7 months (range, 6.4‒32.4 months) for cohort 2, and 22.1 months (range, 8.9‒25.6 months) for cohort 3. Across all 3 cohorts, median time from treatment allocation to data cut off was 22.1 months (range, 4.1‒32.4 months). All patients discontinued study treatment due to PD (n = 12), AEs (n = 2), or physician decision (n = 1; Fig. 1). Median treatment exposure was 2.8 months (range, 0.1‒13.4 months) in cohort 1, 4.5 months (range, 2.2‒7.7 months) in cohort 2, and 3.5 months (range, 3.5‒4.9 months) in cohort 3. Median number of pembrolizumab administrations was 5 (range, 1‒19) in cohort 1, 7 (range, 3‒11) in cohort 2, and 6 (range, 5‒8) in cohort 3. Only 3 patients remained in follow-up when the study was terminated by the sponsor because evaluation of the primary objective had been completed. Overall, 13 of 15 patients received subsequent anticancer therapy after discontinuing study treatment (Online Resource 1).

### Safety

Three patients in cohort 1 experienced a DLT of grade 3 febrile neutropenia during cycle 1. One of these patients also experienced grade 4 laryngeal stenosis that was considered a DLT and discontinued from the study. The other 2 patients received filgrastim and meropenem or cefepime and were able to continue to cycle 2 without delay; the etoposide dose was reduced to 80 mg/m^2^ for both patients, and the cisplatin dose was reduced to 60 mg/m^2^ for 1 patient. No patients in cohort 2 or cohort 3 experienced a DLT.

All 15 patients receiving pembrolizumab in combination with platinum-etoposide chemotherapy experienced ≥ 1 treatment-related AE; the incidence of AEs was similar across all 3 cohorts (Table 2). Discontinuation due to treatment-related AEs was reported in 2 patients (1 each in cohort 1 and cohort 2). No AEs led to death. Serious treatment-related AEs were interstitial lung disease, febrile neutropenia, and laryngeal stenosis (n = 1 each) in cohort 1 and peripheral sensory neuropathy and lichenoid keratosis (n = 1 each) in cohort 2; no serious AEs were reported in cohort 3. Across all cohorts, the most frequently occurring treatment-related AEs included leukopenia, neutropenia, and anemia (Table 2). The most frequently occurring grade 3 or 4 treatment-related AEs were neutropenia, leukopenia, and febrile neutropenia (Table 2).

**Table 2:** Summary of AEs (ATS Population)

**Adverse Event**	**Cohort 1** **Pembrolizumab Plus Cisplatin/Etoposide** **(n = 6)**		**Cohort 2** **Pembrolizumab Plus Carboplatin/Etoposide** **(n = 6)**		**Cohort 3** **Pembrolizumab Plus Cisplatin/Etoposide Plus Pegfilgrastim** **(n = 3)**	
Treatment-related AEs	6 (100)		6 (100)		3 (100)	
Grade 3/4 treatment-related AEs	6 (100)		6 (100)		2 (67)	
Serious treatment-related AEs	3 (50)		2 (33)		0	
Deaths due to a treatment-related AE	0		0		0	
Discontinued due to a treatment-related AE	1 (17)		1 (17)		0	

Treatment-related AEs of any grade occurring in ≥ 2 patients or of grade 3/4 in ≥ 1 patient	**Any Grade**	**Grade 3/4**	**Any Grade**	**Grade 3/4**	**Any Grade**	**Grade 3/4**
Leukopenia	6 (100)	5 (83)	6 (100)	3 (50)	2 (67)	2 (67)
Neutropenia	5 (83)	5 (83)	6 (100)	6 (100)	2 (67)	2 (67)
Constipation	4 (67)	0	1 (17)	0	1 (33)	0
Febrile neutropenia	3 (50)	3 (50)	1 (17)	1 (17)	0	0
Decreased appetite	3 (50)	0	4 (67)	0	2 (67)	0
Alopecia	3 (50)	0	2 (33)	0	2 (67)	0
Nausea	3 (50)	0	2 (33)	0	2 (67)	0
Hyponatremia	2 (33)	1 (17)	1 (17)	1 (17)	0	0
Interstitial lung disease	2 (33)	1 (17)	0	0	0	0
Anemia	2 (33)	0	6 (100)	1 (17)	2 (67)	0
Laryngeal stenosis	1 (17)	1 (17)	0	0	0	0
Thrombocytopenia	1 (17)	0	2 (33)	0	2 (67)	0
Diarrhea	1 (17)	0	1 (17)	1 (17)	1 (33)	0
Pruritus	0	0	3 (50)	0	0	0
Stomatitis	0	0	3 (50)	0	0	0
Lymphopenia	0	0	2 (33)	2 (33)	0	0
Malaise	0	0	2 (33)	0	1 (33)	0
Dysgeusia	0	0	2 (33)	0	0	0
Lichenoid keratosis	0	0	1 (17)	1 (17)	0	0
Peripheral sensory neuropathy	0	0	1 (17)	1 (17)	0	0
Immune-mediated AEs^a^						
Adrenal insufficiency	0	0	0	0	1 (33)	0
Pneumonitis	2 (33)	1 (17)	0	0	0	0
Hypothyroidism	0	0	0	0	1 (33)	0
Hyperthyroidism	0	0	0	0	1 (33)	0

All data are n (%)

*AE* adverse event, *ATS* all treated set

^a^Immune-mediated adverse events and infusion reactions were based on a list of preferred terms intended to capture known risks of pembrolizumab and were considered regardless of attribution to study treatment by the investigator

Immune-mediated AEs were pneumonitis (n = 2) in cohort 1 and adrenal insufficiency, hyperthyroidism, and hypothyroidism (n = 1 each) in cohort 3 (Table 2). The only grade ≥ 3 immune-mediated AE was pneumonitis reported in cohort 1 (Table 2). No grade ≥ 3 immune-mediated AEs were reported in cohort 2 or cohort 3.

### Antitumor activity

**Fig. 2:**
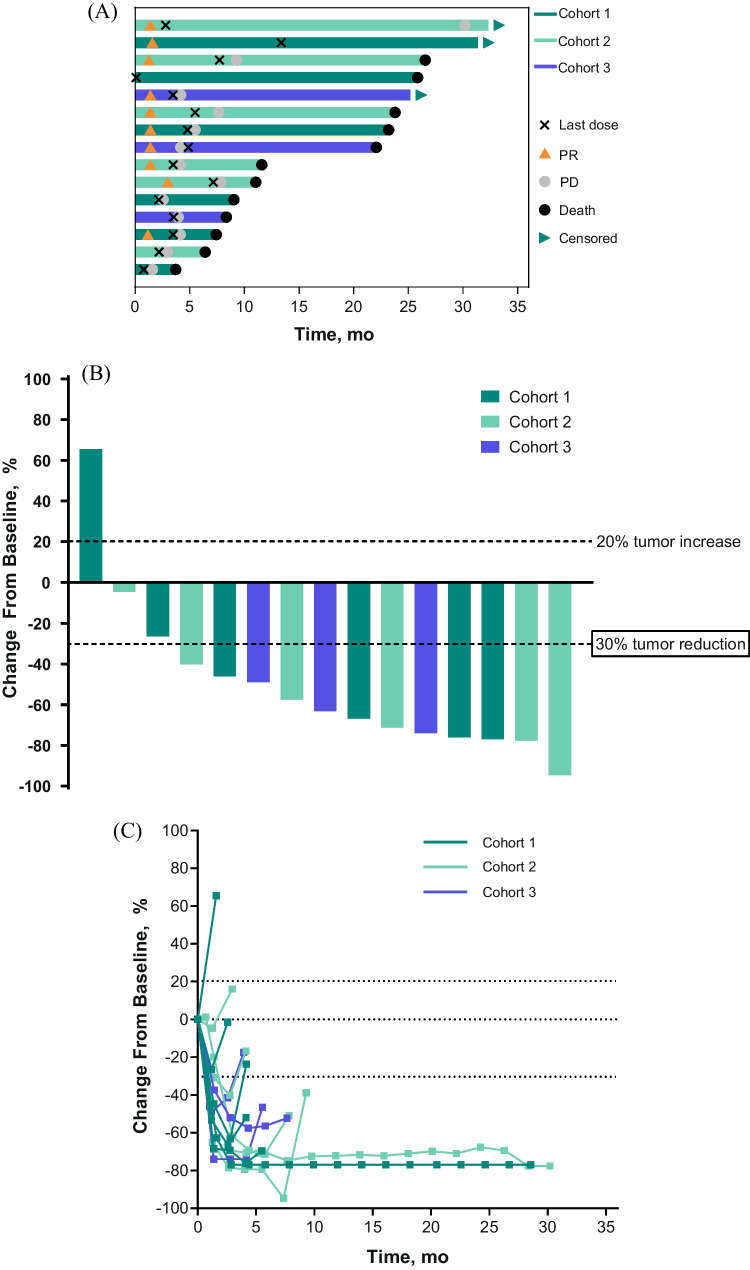
Survival and duration of response in individual patients (**A**), best change in sum of diameters of target lesions from baseline in individual patients (**B**), and percentage change in diameters of target lesions over time in individual patients (**C**). In panel **A**, bar lengths indicate follow-up duration. PD, progressive disease; PR, partial response; SD, stable disease

The ORR across all patients was 67% (95% CI, 38%‒88%). All 10 responses were PRs; no patient experienced a CR (Fig. 2A). The ORR was 50% (95% CI, 12%‒88%) in cohort 1, 83% (95% CI, 36%‒100%) in cohort 2, and 67% (95% CI, 9%‒99%) in cohort 3. Median time to response in all patients was 1.4 months (range, 1.2‒3.0 months) and median DOR was 4.5 months (range, 2.8‒28.8 months). Reductions in tumor size from baseline occurred in 14 of 15 patients (n = 8 in cohorts 1 and 3 combined and n = 6 in cohort 2; Fig. 2B, C).

At the time of data cutoff, 14 of 15 patients (93%) had experienced a PFS event. Across all patients, median PFS was 4.2 months (95% CI, 3.0‒7.8 months), with an estimated PFS rate of 80% (95% CI, 50.0%‒93.1%) at 3 months and 40% (95% CI, 16.5%‒62.8%) at 6 months. Median PFS was 4.8 months (95% CI, 1.6 months‒not reached) for patients in cohort 1, 7.7 months (95% CI, 3.0‒30.2 months) for patients in cohort 2, and 4.2 months (95% CI, 3.9‒4.2 months) for patients in cohort 3.

Twelve of the 15 patients had died by the data cutoff date. Median OS among all patients was 22.1 months (95% CI, 7.4‒25.9 months), with an estimated 12-month survival rate of 53%. Median OS was 16.1 months (95% CI, 3.7 months‒not reached) in patients in cohort 1, 17.7 months (95% CI, 6.4 months‒not reached) in cohort 2, and 22.1 months (95% CI, 8.3 months‒not reached) in cohort 3.

### Discussion

Pembrolizumab in combination with platinum-etoposide chemotherapy was generally well tolerated and showed antitumor activity in Japanese patients with ES-SCLC. DLT rates were 20% (3/15); all patients with a DLT received pembrolizumab in combination with cisplatin and etoposide (ie, cohort 1). Furthermore, this AE profile was generally consistent with findings in a global population receiving pembrolizumab and etoposide combined with either carboplatin or cisplatin for SCLC [14]. As anticipated based on previous reports evaluating pembrolizumab as monotherapy in patients with recurrent or metastatic SCLC [13] or in combination with etoposide-platinum in patients with ES-SCLC [14], previous reports evaluating pembrolizumab, and the immune-mediated mechanism of action for pembrolizumab, immune-mediated AEs were observed, including pneumonitis, adrenal insufficiency, hyperthyroidism, and hypothyroidism. One event of grade 3 pneumonitis occurred in 1 patient in cohort 1; no other grade ≥ 3 immune-mediated AEs were observed.

In this study, the ORR among all patients was 67% (95% CI, 38%‒88%), with a median time to response of 1.4 months (range, 1.2‒3.0 months) and median DOR of 4.5 months (range, 2.8‒28.8 months). While comparison with other studies may be difficult because of the small sample size, these results were consistent with the KEYNOTE-604 study, in which the ORR was 71% (95% CI, 64%‒76%) in the pembrolizumab plus etoposide and platinum group [14]. Among the 15 patients included in the current analysis, nearly all experienced a reduction in tumor size from baseline. Median PFS was 4.2 months (95% CI, 3.0‒7.8 months) and median OS was 22.1 months (95% CI, 7.4‒25.9 months). In the global population enrolled in KEYNOTE-604, pembrolizumab in combination with etoposide and platinum resulted in a similar median PFS of 4.5 months (95% CI, 4.3‒5.4 months) and DOR of 4.2 months (range, 1.0+ to 26.0+ months). However, median OS was 10.8 months (95% CI, 9.2‒12.9 months) in KEYNOTE-604 [14] and thus was markedly shorter than the median OS of 22.1 months (95% CI, 7.4‒25.9 months) in KEYNOTE-011 part E. Results from KEYNOTE-011 part E support the hypothesis that the combination of immune checkpoint inhibitors with chemotherapy provides antitumor activity and can improve outcomes in patients with SCLC.

Limitations of the study include the small number of patients and open-label design. Despite these limitations, the results of this study suggest that the effects of pembrolizumab in a Japanese population are consistent with previous reports in global clinical studies of patients with ES-SCLC.

KEYNOTE-011 part E found that pembrolizumab in combination with platinum-etoposide therapy had manageable toxicity and demonstrated antitumor activity in a Japanese population with ES-SCLC. The current study provides encouraging evidence for immunotherapy targeting the PD-1 pathway in Japanese patients with ES-SCLC. Ongoing studies are evaluating pembrolizumab in combination with chemotherapy and other agents, such as olaparib in patients with limited-stage SCLC (NCT04624204) and as a coformulation with the anti‒T-cell immunoglobulin and ITIM domain inhibitor vibostolimab (TIGIT; NCT05224141), in patients with ES-SCLC.

### Supplementary Information

Below is the link to the electronic supplementary material.

Supplementary file1 (PDF 43 KB)

## Data Availability

Merck Sharp & Dohme LLC, a subsidiary of Merck & Co., Inc., Rahway, NJ, USA (MSD) is committed to providing qualified scientific researchers access to anonymized data and clinical study reports from the company’s clinical trials for the purpose of conducting legitimate scientific research. MSD is also obligated to protect the rights and privacy of trial participants and, as such, has a procedure in place for evaluating and fulfilling requests for sharing company clinical trial data with qualified external scientific researchers. The MSD data sharing website (available at: http://engagezone.msd.com/ds_documentation.php) outlines the process and requirements for submitting a data request. Applications will be promptly assessed for completeness and policy compliance. Feasible requests will be reviewed by a committee of MSD subject matter experts to assess the scientific validity of the request and the qualifications of the requestors. In line with data privacy legislation, submitters of approved requests must enter into a standard data-sharing agreement with MSD before data access is granted. Data will be made available for request after product approval in the US and EU or after product development is discontinued. There are circumstances that may prevent MSD from sharing requested data, including country or region-specific regulations. If the request is declined, it will be communicated to the investigator. Access to genetic or exploratory biomarker data requires a detailed, hypothesis-driven statistical analysis plan that is collaboratively developed by the requestor and MSD subject matter experts; after approval of the statistical analysis plan and execution of a data-sharing agreement, MSD will either perform the proposed analyses and share the results with the requestor or will construct biomarker covariates and add them to a file with clinical data that is uploaded to an analysis portal so that the requestor can perform the proposed analyses.
