# Supplementary material for: Phase 1 study of pembrolizumab plus chemotherapy in Japanese patients with extensive-stage small-cell lung cancer
Source: Invest New Drugs. 2024 Feb 1;42(1):136–44. doi: 10.1007/s10637-023-01411-1 (PMC10891246; doi:10.1007/s10637-023-01411-1)
Supplement: Supplementary file 1 — Supplementary file1 (PDF 43 KB) [file 10637_2023_1411_MOESM1_ESM.pdf]

SUPPLEMENTAL MATERIAL

Phase 1 Study of Pembrolizumab Plus Chemotherapy in Japanese Patients With Extensive-Stage Small-Cell Lung Cancer

Naoyuki Nogami,[1] Takaaki Tokito,[2] Yoshitaka Zenke,[3] Miyako Satouchi,[4] Takashi Seto,[5] Hideo Saka,[6] Junko Otani,[7] Shirong Han,[7] Kazuo Noguchi,[7] Makoto Nishio[8]

[1]Ehime University Graduate School of Medicine, Department of Community Medicine, Pulmonology and Cardiology, Toon, Japan; [2]Division of Respirology, Neurology, and Rheumatology, Department of Internal Medicine, Kurume University School of Medicine, Kurume, Japan; [3]Department of Thoracic Oncology, National Cancer Center Hospital East, Kashiwa, Japan; [4]Department of Thoracic Oncology, Hyogo Cancer Center, Akashi, Japan; [5]Department of Thoracic Oncology, National Hospital Organization Kyushu Cancer Center, Fukuoka, Japan; [6]Department of Respiratory Medicine, National Hospital Organization Nagoya Medical Center, Nagoya, Japan; [7]MSD K.K., Tokyo, Japan; [8]Department of Thoracic Medical Oncology, The Cancer Institute Hospital of Japanese Foundation for Cancer Research, Tokyo, Japan

Corresponding Author: Naoyuki Nogami
Department of Community Medicine, Pulmonology and Cardiology
Ehime University Graduate School of Medicine
454 Shitsukawa, Toon, Ehime 791-0295
Japan
Email: nogami.naoyuki.zx@ehime-u.ac.jp

Table S1. Subsequent anticancer therapy

Subsequent therapy	Second line (n = 13)	Third line (n = 8)	Fourth line (n = 5)
Amrubicin	10	3	0
Cisplatin plus etoposide	1	0	0
Carboplatin plus etoposide	1	0	0
Investigational drug	1	1	0
Topotecan	0	2	0
Cisplatin plus irinotecan	0	2	0
Eribulin	0	0	2
Carboplatin plus paclitaxel	0	0	1
Erlotinib	0	0	1
Atezolizumab, carboplatin, and etoposide	0	0	1
